# Supplementary material for: A Global Screen for Assembly State Changes of the Mitotic Proteome by SEC-SWATH-MS
Source: Cell Syst. 2020 Feb 26;10(2):133–155.e6. doi: 10.1016/j.cels.2020.01.001 (PMC7042714; doi:10.1016/j.cels.2020.01.001)

Q13445 | TMED1_HUMAN | TMED1 IL1RL1L IL1RL1LG
Monomer MW [kDa]: 25.206 Monomer expected elution fraction: 52

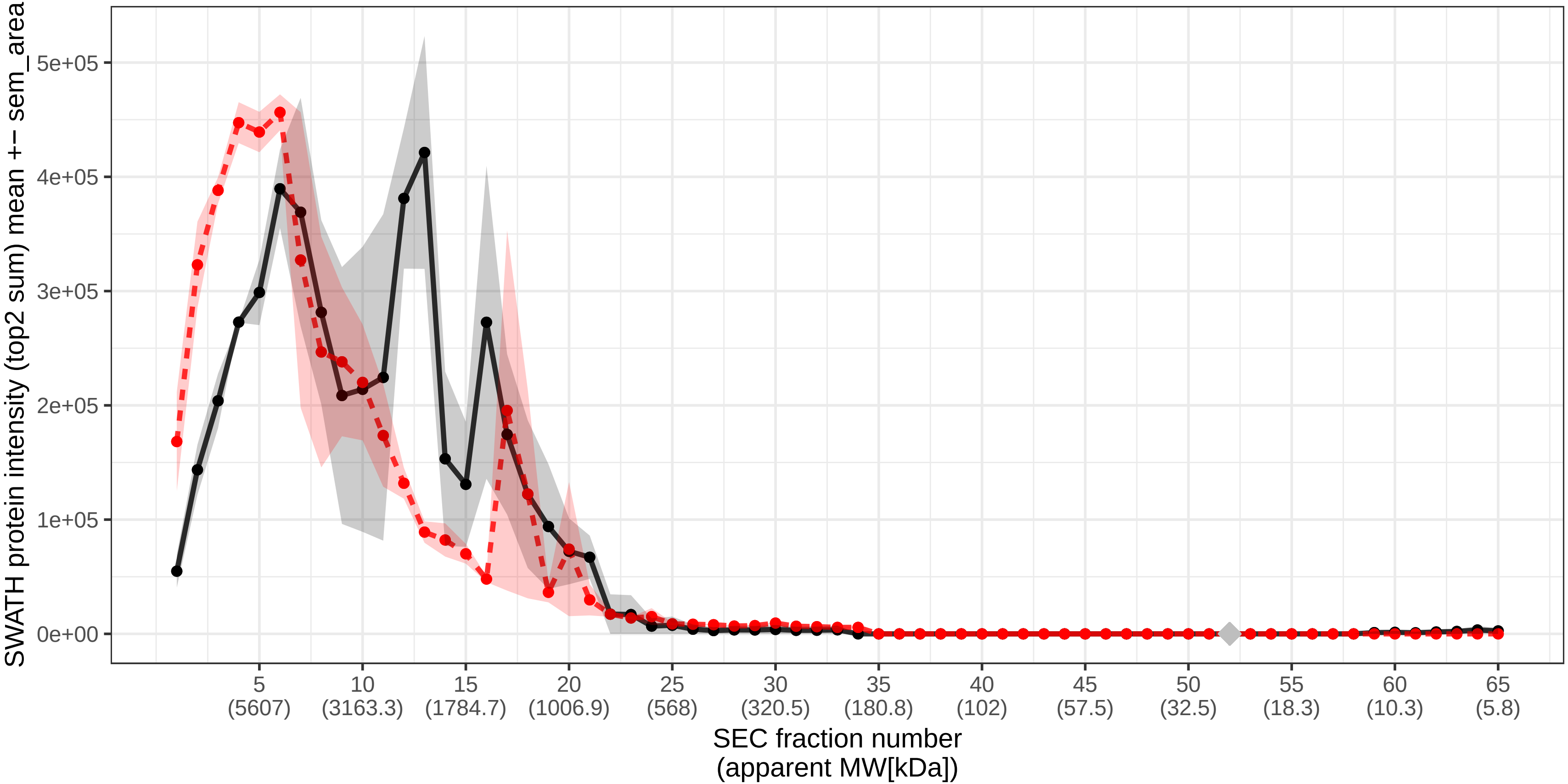

Supplement: Data S1. SEC-SWATH-MS Protein Chromatograms, Related to Figure 1 [file mmc6.zip › SECchrom_Q13445_TMED1_HUMAN_TMED1_IL1RL1L_IL1RL1LG.pdf]
